# Long-term course of phrenic nerve injury after cryoballoon ablation of atrial fibrillation

**DOI:** 10.1038/s41598-021-85618-3

**Published:** 2021-03-18

**Authors:** Michifumi Tokuda, Seigo Yamashita, Hidenori Sato, Hirotsuna Oseto, Hirotsugu Ikewaki, Masaaki Yokoyama, Ryota Isogai, Ken-ichi Tokutake, Ken-ichi Yokoyama, Mika Kato, Ryohsuke Narui, Shin-ichi Tanigawa, Seiichiro Matsuo, Michihiro Yoshimura, Teiichi Yamane

**Affiliations:** Department of Cardiology, The Jikei University School of Medicine, 3-25-8, Nishi-shinbashi, Minato-ku, Tokyo, 105-8461 Japan

**Keywords:** Interventional cardiology, Atrial fibrillation

## Abstract

While phrenic nerve palsy (PNP) due to cryoballoon pulmonary vein isolation (PVI) of atrial fibrillation (AF) was transient in most cases, no studies have reported the results of the long-term follow-up of PNP. This study aimed to summarize details and the results of long-term follow-up of PNP after cryoballoon ablation. A total of 511 consecutive AF patients who underwent cryoballoon ablation was included. During right-side PVI, the diaphragmatic compound motor action potential (CMAP) was reduced in 46 (9.0%) patients and PNP occurred in 29 (5.7%) patients (during right-superior PVI in 20 patients and right-inferior PVI in 9 patients). PNP occurred despite the absence of CMAP reduction in 0.6%. The PV anatomy, freezing parameters and the operator’s proficiency were not predictors of PNP. While PNP during RSPVI persisted more than 4 years in 3 (0.6%) patients, all PNP occurred during RIPVI recovered until one year after the ablation. However, there was no significant difference in the recovery duration from PNP between PNP during RSPVI and RIPVI. PNP occurred during cryoballoon ablation in 5.7%. While most patients recovered from PNP within one year after the ablation, PNP during RSPVI persisted more than 4 years in 0.6% of patients.

## Introduction

Cryoballoons have proven to be effective for pulmonary vein isolation (PVI) in patients with atrial fibrillation (AF). Several recent randomized trials have shown the noninferiority of cryoballoon ablation to radiofrequency ablation with respect to the treatment efficacy in patients with drug-refractory paroxysmal AF^1, 2^.

Phrenic nerve palsy (PNP) is the most frequently observed complication during cryoballoon ablation. Since the cryoballoon is not variable, it is difficult to freeze PV away from the position of the phrenic nerve. Thus, the incidence of PNP was reported higher in comparison to that during radiofrequency catheter ablation^1–5^. Several methods for the early detection of PNP have been reported^6^. Although, PNP is usually transient with complete resumption of right diaphragmatic contraction before the end of ablations, PNP persisted in some patients. However, the long-term course of cryothermal PNP have not well been determined. The purpose of this study was to investigate the long-term consequence of PNP after cryoballoon ablation.

## Methods

### Study subjects

A total of 511 consecutive patients who underwent cryoballoon ablation for AF were included in the present study. Paroxysmal AF was defined as AF that spontaneously terminated within seven days. Antiarrhythmic drugs were discontinued for at least five half-lives prior to ablation. A 3D image of the PV was reconstructed by preprocedural enhanced computed tomography. The size and length of the PV trunk (between the ostium and the first branch) were measured by an electro-anatomical mapping system^7^.

### Ethics

Clinical investigations were conducted in accordance with the principles expressed in the Declaration of Helsinki. All data were compliant with the International Conference on Harmonization guidelines. All experimental protocols were approved by The Ethical Committee of the Jikei University School of Medicine. All methods were carried out in accordance with relevant guideline and regulations. The informed consent was obtained from all participants.

### Cryoballoon ablation of AF

Details of cryoballoon ablation for AF and early detection of phrenic nerve injury were reported as previously described^8–10^. A single transseptal puncture was performed using a radiofrequency needle (Baylis Medical, Montreal, QC, Canada) and an 8.5-Fr long sheath (SL0; Abbott, Chicago, IL). The transseptal sheath was exchanged over a guidewire for a 15-Fr steerable sheath (Flexcath Advance; Medtronic, Minneapolis, MN). Another SL0 sheath was inserted to the left atrium via the same puncture site and a circumferential 20-pole catheter (Lasso 2515 NAV eco variable catheter; Biosense Webster, Diamond Bar, CA) was inserted to map all PVs before and after the cryoballoon to confirm electrical isolation. PVI was performed with a single balloon technique using a second-generation (Arctic Front Advance; Medtronic) or 4^th^-generation cryoballoon (Arctic Front Advance Pro; Medtronic). A 28-mm cryoballoon catheter was used in all of the patients. A spiral mapping catheter (Achieve; Medtronic) was used to advance the cryoballoon and map the PV potentials. Complete sealing at the antral aspect of the PV was confirmed by the injection of contrast medium. The proximal-seal technique was used, if possible. This was followed by a freeze cycle of 180 s. In 100 patients, a 120-s bonus-freeze was applied after the successful application of a 180-s initial freeze. If electrical isolation was not achieved by cryoballoon, additional touch-up ablation was performed with a conventional radiofrequency or cryothermal (Freezer Max; Medtronic) catheter.

### Early detection of phrenic nerve injury

To avoid phrenic nerve injury, the diaphragmatic compound motor action potentials (CMAPs) were monitored during right phrenic nerve pacing, as previously described^6, 11, 12^. A standard decapolar catheter or a circumferential 20-pole catheter was placed in the superior vena cava (SVC) cranial to the RSPV in order to pace the PN, in recent cases, the circular catheter was generally positioned in the subclavian vein in order to achieve better stability and a more reliable PN capture in comparison to the SVC. For the early detection of PNP, the pacing threshold was measured, and the pacing output was set slightly above the pacing threshold. If a 30% reduction of CMAP or a loss of capture was observed, the freeze was immediately aborted using a double-stop technique^13^ and observed for recovery. Additional cryo-applications were not performed even if PNI recovered during the procedure. CMAP monitoring between left side PVI was not performed.

### Patient follow-up

Details of patient follow-up were reported as previously described^8–10^. In patients with paroxysmal AF, no antiarrhythmic drugs were prescribed after the procedure. The patients underwent continuous, in-hospital ECG monitoring for 2–4 days after the procedure. The patients underwent careful observation (two weeks after discharge, then every month thereafter) at the cardiology clinic. The outcome of AF ablation was evaluated based on the patient’s symptoms, ECG at periodical follow-up examinations, and periodic 24-h ambulatory monitoring (at 1, 3, 6, 9, 12 months and yearly after the procedure). The recurrence of AF was defined as AF lasting for more than 30 s after a blanking period of 90 days.

### Impending, transient and persistent PNP

The degree of PNP was divided into three grades. A > 30% reduction in CMAP without weakening of diaphragmatic motility was considered as impending PNP. Definition of transient and persistent PNP was previously described^14^. Specifically, transient PNP was defined as a progressive weakening of diaphragmatic motility, as assessed by manual palpation on the abdomen, confirmed by fluoroscopy or the occurrence of a hemidiaphragm paralysis detected by both manual palpation and fluoroscopy during the procedure, and completely resolving before the end of the procedure. Persistent PNP was defined as an elevated hemidiaphragm noted on post-procedural radiography, which persisted after the procedure. The diaphragmatic function on a chest X-ray was confirmed by a radiologist based on the shape of an elevated hemidiaphragm in both postero-anterior and in lateral fluoroscopy projections during a sniff maneuver. The position and shape of the diaphragm were determined by correlating measurements to skeletal structures and the radius of the curvature, respectively. Once the diagnosis of PNP was established, the patient was closely monitored in the clinic with tests repeated every 3 months. Complete recovery of the phrenic nerve function was diagnosed in the case of normalization of the diaphragm position in X-ray images both at rest and during a sniff test by comparison with the pre-procedural chest images.

### Statistical analyses

Categorical variables were analyzed using a chi-squared test, unless the expected values in any cells were < 5, in which case Fisher’s exact test was used. *P*-values of < 0.05 were considered to indicate statistical significance. Continuous variables were expressed as the mean ± standard deviation. A Mann–Whitney U test or unpaired Student’s *t*-test was used for the analysis of continuous variables. The Kaplan–Meier method were used for survival curve analysis and comparisons between groups were performed using a log-rank test. All statistical analyses were performed using the SPSS software program (version 27; SPSS, Chicago, IL, USA).

## Results

### Pulmonary vein isolation

A total of 511 patients who underwent cryoballoon ablation for AF were included in this study. The baseline characteristics are shown in Table 1. The mean age and left atrial diameter were 59.8 ± 10.1 years and 37.9 ± 16.3 mm, respectively. Cryoballoon isolation was performed in the LSPV (n = 511), LIPV (n = 511), RSPV (n = 506) and RIPV (n = 495). In right-side PV, the PV was isolated in the order of the RSPV to the RIPV in 369 patients and the RIPV to the RSPV in 142 patients. Touch-up ablations were required 5.5% in LSPV, 6.8% in LIPV, 2.3% in RSPV and 22% in RIPV. Isolation of the SVC was performed in 61 (12%) patients by RF catheter.

**Table 1:** Patient characteristics.

	N = 511	PNP (−) N = 482	PNP (+) N = 29	*P* value
Sex (male)	423 (83%)	403 (84%)	20 (69%)	0.04
Age (years)	59.8 ± 10.1	59.3 ± 10.8	62.9 ± 9.5	0.59
Body mass index (kg/m^2^)	24.1 ± 3.2	24.1 ± 3.3	24.2 ± 3.4	0.93
Left atrial diameter (mm)	37.9 ± 16.3	37.9 ± 17.0	36.4 ± 5.4	0.25
LVEF (%)	64.4 ± 5.8	64.3 ± 5.8	64.1 ± 4.4	0.78
eGFR (ml/min/1.73 m^2^)	76.9 ± 17.0	76.7 ± 17.3	77.8 ± 13.8	0.81
BNP (pg/ml)	56.1 ± 72.5	56.1 ± 72.5	49.8 ± 45.7	0.66
Hypertension	207 (41%)	194 (40%)	13 (45%)	0.63
Diabetes mellitus	52 (10%)	50 (10%)	2 (7%)	0.55
CHADS_2_ score	0.7 ± 0.9	0.7 ± 0.9	0.7 ± 0.8	0.87
CHA_2_DS_2_-VASc score	1.2 ± 1.2	1.2 ± 1.3	1.4 ± 1.1	0.45
Paroxysmal AF	482 (94%)	457 (95%)	25 (86%)	0.052
**Operator’s experience (cases)**				
< 20 cases	153 (30%)	149 (30%)	4 (22%)	0.60
< 30 cases	205 (40%)	199 (41%)	6 (33%)	0.54
< 40 cases	255 (50%)	247 (50%)	8 (44%)	0.62
< 50 cases	296 (58%)	286 (58%)	10 (56%)	0.81

The data are presented as the mean ± standard deviation.

*LVEF* left ventricular ejection fraction, *eGFR* estimated glomerular filtration rate, *BNP* B-type natriuretic peptide, *AF* atrial fibrillation.

### Phrenic nerve injury during cryoballoon procedure

During right-side PVI, the CMAP was reduced in 46 (9.0%) patients. PNP occurred during a cryoballoon procedure in 29 (5.7%) patients (4.0% [20/506] in the RSPV and 1.8% [9/495] in the RIPV). Among these cases, CMAP reduction preceded PNP in 26 (5.1%) patients (3.6% [18/506] in the RSPV and 1.6% [8/495] in the RIPV). On the other hand, PNP occurred despite the absence of CMAP reduction during the procedure in 3 (0.6%) patients. None of these 3 patients underwent SVC isolation and/or RF touch-up ablation (Table 2). The incidence of PNP in men was higher than that in women (84% vs. 69%, *P* = 0.04, Table 1).

**Table 2:** PNP without CMAP reduction.

	Patient A	Patient B	Patient C
Age (years)	72	59	55
Gender	Female	Male	Male
PV	RSPV	RIPV	RSPV
Time to isolation (sec)	40	57	36
Total freezing time (sec)	180	180	180
Freezing time after PV isolation (sec)	140	123	144
Nadir temperature (°C)	− 52	− 50	− 47
Baseline CMAP amplitude (mV)	0.25	0.48	0.50
Min CMAP amplitude (mV)	0.21	0.39	0.42
CMAP reduction	16%	19%	16%
Pacing catheter	Circular	Circular	Circular
Pacing site	Subclavian vein	Subclavian vein	Subclavian vein
Touch up ablation	(−)	(−)	(−)
Days to recovery	16	10	21

*RSPV* right superior pulmonary vein, *RIPV* right inferior pulmonary vein, *CMAP* compound motor action potential.

The details of freezing application in the RSPV and RIPV (Table 3) were compared between patients with and without PNP. In both RSPV and RIPV, the number of freezing applications was smaller, and the total freezing time was shorter in the patients with PNP than in those without.

**Table 3:** Procedural details.

	RSPV			RIPV		
	PNP (−) n = 486	PNP (+) n = 20	*P*	PNP (−) n = 486	PNP (+) n = 9	*P*
4th generation cryoballoon	23 (4.7%)	1 (5.0%)	0.96	23 (4.8%)	2 (25%)	0.07
No. of freezing	1.4 ± 0.6	1.1 ± 0.4	0.001	1.5 ± 0.8	1.1 ± 0.3	0.13
Time to − 30 °C	26.5 ± 5.3	26.8 ± 3.5	0.81	31.7 ± 9.8	31.1 ± 4.9	0.76
Time to − 40 °C	43.7 ± 13.0	45.0 ± 10.5	0.71	62.0 ± 35.1	52.9 ± 13.9	0.17
Time to isolation (sec)	34.0 ± 22.5	31.2 ± 18.8	0.56	45.5 ± 30.3	68.5 ± 16.3	0.29
Total freezing time (sec)	220 ± 76	151 ± 64	< 0.001	233 ± 101	144 ± 45	< 0.001
Nadir balloon temperature (°C)	− 54.4 ± 5.5	− 52.3 ± 3.8	0.12	− 49.0 ± 7.6	− 48.1 ± 6.2	0.67
Circumferential length of PV ostium (mm)	59.3 ± 12.3	63.5 ± 17.1	0.35	53.4 ± 12.0	49.9 ± 6.7	0.16
Length of PV trunk (mm)	18.9 ± 7.1	19.0 ± 6.8	0.97	13.0 ± 5.1	11.9± 3.6	0.38
The order of PVI (RIPV → RSPV)	131 (28%)	6 (30%)	0.76	133 (27%)	4 (44%)	0.27
**Phrenic nerve pacing for CMAP recording**						
Pacing from the subclavian vein	409 (84%)	19 (95%)	0.18	411 (85%)	8 (89%)	0.72
Paced by the circular catheter	138 (28%)	8 (40%)	0.26	141 (29%)	7 (78%)	0.004
Pacing threshold (V)	2.9 ± 1.6	2.2 ± 0.9	0.09	2.9 ± 1.6	2.5 ± 0.7	0.62
Maximum CMAP reduction (%)	3.0 ± 21.1	39.1 ± 40.6	0.01	0.8 ± 22.8	36.6 ± 48.0	0.008

The data are presented as the mean ± standard deviation.

Abbreviations are the same as in the previous table.

The nadir balloon temperature, time to isolation, time to  − 30 °C, time to  − 40 °C, circumferential length of the PV ostium and length of the PV trunk were similar between two groups. Cryoballoon ablation was performed by 8 operators in this study. Incidence of PNP was similar among 8 operators (*P* = 0.57, range 0–9.1%). Incidence of PNP was not different between the first 20 cases of the operator and subsequent cases (3.4% vs. 3.8%, *P* = 0.81 in RSPV and 1.0% vs 2.4%, *P* = 0.23 in RIPV).

### Follow up

**Figure 1:**
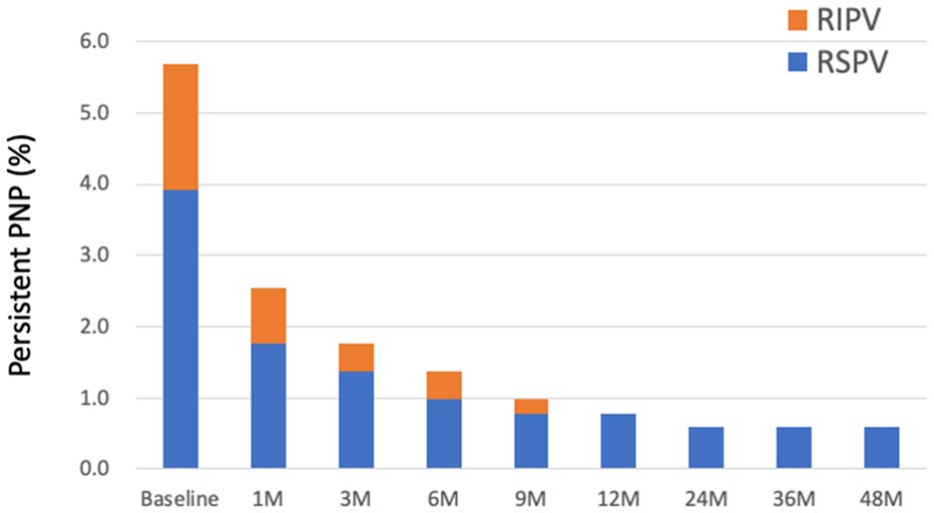
The incidence of persistent PNP after the procedure.

**Figure 2:**
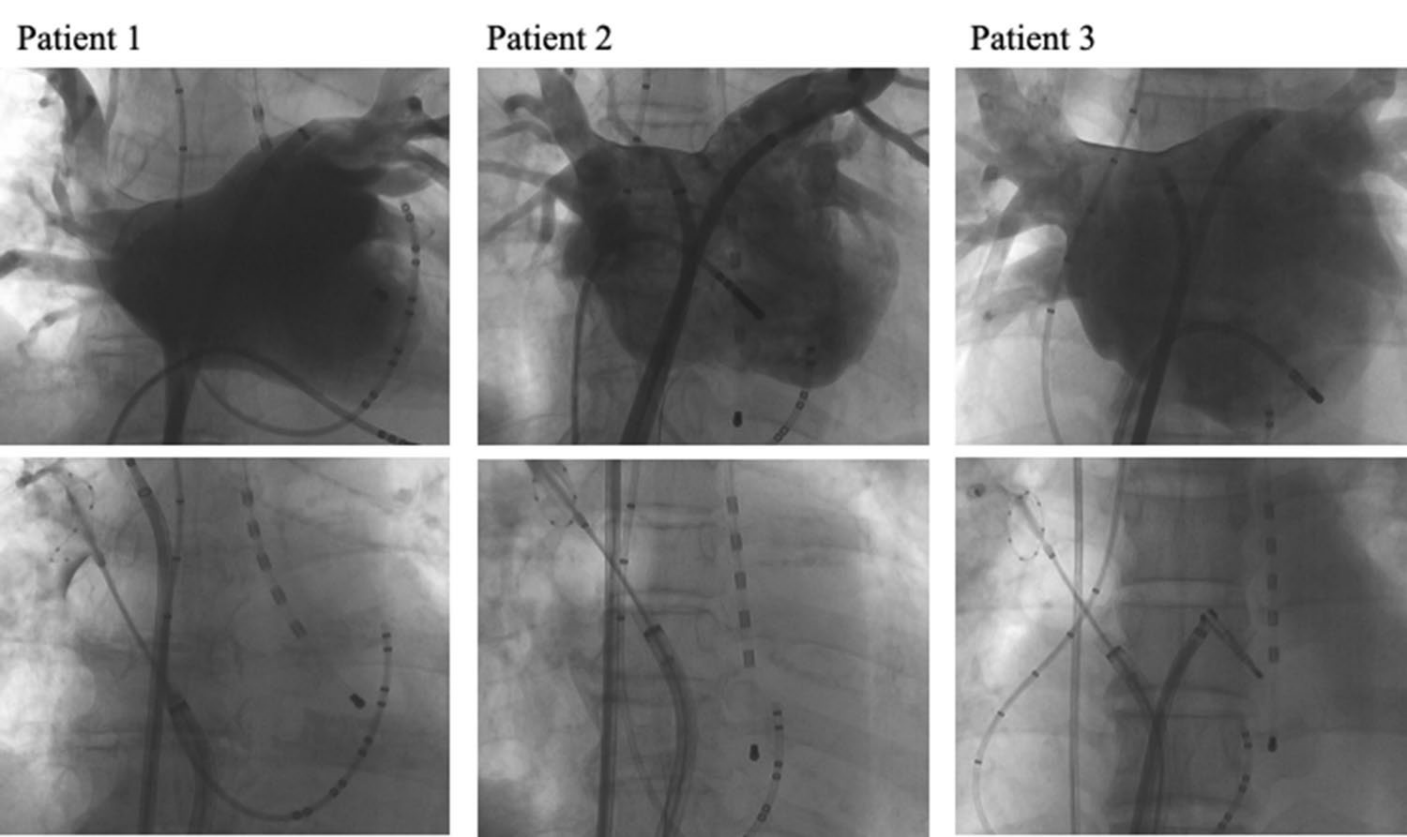
PV angiography and balloon position on the fluoroscopy in patients with persistent PNP during RSPV applications (anteroposterior view). Deep balloon position due to the large RSPV ostium was shown in patient 2 and 3. PV indicates pulmonary vein; PNP, phrenic nerve injury; and RSPV, right superior pulmonary vein.

In three patients, PNP recovered at the end of the procedure. AF free rate was similar between patients with PNP and those without (*P* = 0.82). No patients complained any symptoms regarding as PNP. At one years after the procedure, 25 of 29 patients had recovered from PNP. On the other hand, PNP persisted more than 4 years in 3 (0.6%) patients (Fig. 1). In all three patients, PNP occurred during freezing of the RSPV. The balloon position on the fluoroscopy (Fig. 2) and procedural parameters (Table 4) of these patients were shown. The mean nadir temperature and time to isolation were  − 52 °C and 25 s, respectively. In patient 2 and 3, deep balloon position during RSPV application was shown during freezing of the RSPV. SVC isolation and touch-up RF ablation was not performed in these 3 patients.

**Table 4:** Procedural details (patients with persistent PNP).

	Patient 1	Patient 2	Patient 3
Age (years)	75	68	47
Gender	Female	Female	Male
Time to isolation (sec)	–	24	26
Total freezing time (sec)	171	83	118
Nadir temperature (°C)	− 52	− 48	− 57
Maximum CMAP reduction (%)	24	42	66
Stop method	Double stop	Double stop	Double stop
Touch up ablation	None	None	None
SVC isolation	None	None	None
Circumferential length of RSPV (mm)	61	65	No CT
Length of RSPV trunk (mm)	11	11	No CT
Fluoroscopic position of cryoballoon in relation to the cardiac shadow	None	Distal 1/3	Mid 1/3
Operator’s experience (cases)	55	7	53

*SVC* superior vena cava.

The position of cryoballoon was evaluated under fluoroscopic guidance in AP projection during RSPV ablation. None: the lower half of the balloon is completely located inside the cardiac shadow. Distal 1/3: less than one-third of the lower portion of the balloon is located outside the cardiac shadow. Mid 1/3: one-third or more of the lower portion of the balloon is located outside the cardiac shadow.

All PNPs that occurred during RIPV isolation recovered within one year after ablation. The duration of recovery from PNP was similar between patients who developed PNP during freezing of the RSPV and those who developed PNP freezing of the RIPV in survival analysis (log rank *P* = 0.21). The median time to recovery from PNP after ablation was 30 days in the RSPV and 39 days in the RIPV.

### Repeat ablation procedure

In 46 patients with CMAP reduction during PV application, repeat ablation procedure was performed in 4 patients (RSPV in 3, RIPV in 1). Of those, PV reconnection was observed in 67% (2/3) of the RSPV and none of the RIPV (Table 5). While freezing time after the elimination of RSPV potential was 51 s and 55 s in the patient with RSPV reconnection, it was 166 s in the patient without RSPV reconnection. Reconnected site was located in anterior wall in one patient and both anterior and posterior wall in another patient.

**Table 5:** Repeat ablation procedure.

	Patient I	Patient II	Patient III	Patient IV
Age (years)	56	45	61	60
Gender	Male	Male	Female	Male
PV	RSPV	RSPV	RIPV	RSPV
Time to isolation (sec)	33	18	–	14
Total freezing time (sec)	84	73	157	180
Freezing time after PV isolation (sec)	51	55	–	166
Nadir temperature (°C)	− 49	− 50	− 54	− 56
Stop method	Double stop	Double stop	Double stop	Double stop
Touch up ablation	(−)	(−)	(+)	(−)
PV reconnection	(+)	(+)	(−)	(−)
PV reconnection site	Anterior	Anterior, posterior	N/A	N/A

Abbreviations are the same as in the previous tables.

## Discussion

### Phrenic nerve palsy

This study reports the consequences of patients with PNP during cryoballoon PVI with a long-term follow-up period (> 4 years). This is the first study to use a survival analysis to compare the consequences of PNP occurring during RSPVI to PNP occurring during RIPVI. PVI using cryoballoon was performed for the LSPV (n = 511), LIPV (n = 511), RSPV (n = 506) and RIPV (n = 495), respectively. When PNP occurs in either the RSPV or RIPV, the remaining PVI was not performed by the cryoballoon. Thus, there was a difference in the number of cases among 4 PVs. PNP occurred in 5.7% of patients. This is consistent with previous reports^1, 4, 5, 14, 15^. However, this rate was higher than that in recent studies reporting on data obtained using contemporary techniques^11, 16, 17^. The incidence of PNP can be decreased by increased operator experience to avoid deep seating of the cryoballoon in PV, and improved techniques that allow the early detection of PNP. However, incidence of PNP was not related to the operator’s proficiency in this study.

The PN runs in front of the distal right PV; thus, cooling at a deeper position increases the risk of PNP^4^. In previous studies, the distance from the RSPV ostium to the right peri-cardiophrenic bundles^15^, the larger RSPV ostial area^5, 11^ and the external RSPV-left atrium angle^5^ were predictors of PNP during cryoballoon PVI. In this study, the time to  − 30 °C, time to  − 40 °C and the nadir balloon temperature did not differ to a statistically significant extent and the PV size and length of the PV trunk measured by preoperative CT did not differ between the two groups to a statistically significant extent.

PNP occurred even during RIPVI in 1.6% of patients. In a previous study, the incidence of PNP during cryoballoon RIPVI was 3.5%^14^. The authors reported that the velocity of the temperature drop from basal to  − 20 °C and the presence of a right common ostium were predictors of PNP. In our institute, cryoballoon was not used in cases involving a common pulmonary vein trunk. The proximal-seal technique to better define the PV ostium and ensure a proximal ablation lessened the risk for PNP at RSPV^12^. However, this technique often difficult to apply during the RIPVI. No patients experienced irreversible PNP during freezing of the RIPV. The right PN descends parallel along the anterolateral aspect of the SVC, and then courses posteriorly between the RSPV and the SVC. Histological studies have shown that there may be as little as 2.1 mm (± 0.4 mm) and 7.8 mm (± 1.2 mm) of distance between the PN and the RSPV and RIPV, respectively^18^. Cryothermal energy can create lesions to depths of 2 to 5 mm^19^. This close proximity accounts for the higher risk of both transient and persistent PNP during RSPVI than RIPVI. PNP during RIPVI was more frequent when PN pacing was performed using a circular catheter. It is considered that there were some other intervening factors such as when the ablation was performed. However, since the number of patients with PNP during RIPVI was small, it is difficult to perform a multivariate analysis. Further larger-scale studies are needed to reveal the predictors of PNP during RIPVI.

PNP occurred in 3 cases without CMAP reduction during freezing. It is unclear when it occurred. In these cases, PNP may have occurred during the thawing time or the onset may have been late. All 3 patients recovered from the PNP within one month after the procedure. PN pacing and CMAP recording should be continued during thawing until cryoballoon deflation.

### Time-course of recovery and irreversible PNP

The long-term outcomes of the STOP AF PAS study demonstrated that the rate of PNP was 3.2%. PNP resolved within 36 months in all but 1 of the patients^17^. However, the rate was not separately evaluated in the RSPV and RIPV. The results of this study showed that a median duration of recovery from PNP was 30–40 days and most patients recovered from PNP within one year after the ablation procedure. On the other hand, none of PNP recovered after the second year. In all three of these patients, PNP occurred during freezing of the RSPV. However, a survival analysis showed no significant difference in the duration of recovery from PNP between the RSPV and RIPV. No left PNP occurred in this study. A previous study reported on cases with left PNP and recommended the performance of PN pacing during left PVI^20^.

### Mechanism of PNP during cryoballoon application

Since the cryoballoon is not variable, it is difficult to change the site of the freezing application and higher rates of PNP have been reported in comparison to other ablation systems. The incidence of PNP during RF ablation was reported to be 0.3%^3^. While the incidence of PNP during cryoballoon PVI was higher than that during RF ablation, PNP was transient in most cases. Since the ion channel function is temperature-dependent, cryoablation in close proximity to adjacent nerves can result in focal cold-induced conduction block. In a previous study^21^, large axonal loss was observed as a histological change during cryothermal PNP. Wallerian degeneration associated with the subsequent regeneration of axons was observed in most cases. Since the extent of damage from cryoballoon applications was proportional to the temperature changes and the total amount of energy delivered, the early detection of PNP by observing CMAP reduction could prevent the aggravation of PNP. However, the number of freezing applications and total freezing time were shorter in patients with PNP compared to those without in this study. This was likely a result of operators urgently terminating the freezing application when CMAP reduction or a loss of the PN capture occurred.

In patients with CMAP reduction during RPV application, repeat ablation procedure was performed in three patients. Of those, RSPV was reconnected in 2(67%) patients. In two reconnected RSPV, freezing time after PV potential elimination was shorter than the PV without reconnection. The durability of PV isolation was lower for PVs with earlier double stop due to CMAP reduction. A previous study showed 50% of RSPVs were reconnected during the second procedure^11^. They also conclude that 1-min freezes seem to be insufficient, however, 2-min freezes might be sufficient to obtain good durability.

The results of this study indicated that when careful observation was performed to detect CMAP reduction, the incidence of irreversible PNP was low. In this study, no patient with PNP reported experiencing symptoms.

Several limitations associated with the present study warrant mention. This study was a non-randomized, observational, single-center study. Since respiratory function tests were not performed in this study, we could not assess the severity of PNP and influence on the respiratory function and it is unclear whether the respiratory function fully recovered. The relative health and preserved functional capacity at baseline may relate that all patients with PNP were asymptomatic. Moreover, patients in the current study underwent preprocedural MDCT and those with non-conventional anatomy were not offered cryoballoon ablation.

## Conclusions

PNP occurred in 5.7% of patients after cryoballoon ablation for AF. PNP even occurred during RIPVI in 1.8% of patients. The anatomy of PV, freezing parameters and the operator’s proficiency were not predictors of PNP. While all PNP due to RIPVI recovered within one year after the ablation, persistent PNP more than 4 years due to RSPVI was observed in 0.6% of patients. However, there was no significant difference in the recovery duration from PNP between PNP during RSPVI and RIPVI.
